# Cardiopulmonary bypass via common carotid artery cannulation in redo sternotomy

**DOI:** 10.1186/1749-8090-2-31

**Published:** 2007-07-05

**Authors:** Sunil K Bhudia, Hunaid A Vohra, Asif Hassan, Qamar Abid

**Affiliations:** 1 Department of Cardiothoracic Surgery University Hospital of North Staffordshire NHS Trust, Stoke-on-Trent, UK; 2 Department of Cardiac Surgery, Freeman Hospital, Newcastle-upon-Tyne, UK

## Abstract

There are certain situations in redo cardiac surgery in adults where it may not be possible to use alternate arterial cannulation sites like the common femoral artery and axillary artery. We report a case where we established safe cardiopulmonary bypass with common carotid artery cannulation in an adult patient. The patient underwent aortic valve replacement for severe aortic regurgitation 8 months after repair of type A aortic dissection plus aortic valve resuspension.

## Case Report

A 55 year old gentleman underwent emergency ascending aortic replacement and aortic valve repair (valve resuspension) for a type A aortic dissection. He made an excellent recovery following this procedure and postoperative transthoracic echocardiogram (TTE) showed mild aortic valve regurgitation. A TTE repeated 4 weeks later showed presence of moderate aortic valve regurgitation. Follow-up investigations at six months showed that the ascending aorta repair was intact and the dissection flap extending to the left common iliac artery with minimal flow in the false lumen. At this stage there was no change in the degree of aortic regurgitation. However, cardiac catheterisation was performed at 8 months which revealed severe aortic regurgitation, systolic pulmonary artery pressure of 51 mmHg and impaired left ventricular function. Gradual failure of the aortic valve repair to resuspend the valve was speculated to be the likely cause of significant aortic regurgitation. Aortic valve replacement was contemplated.

**Figure 1:**
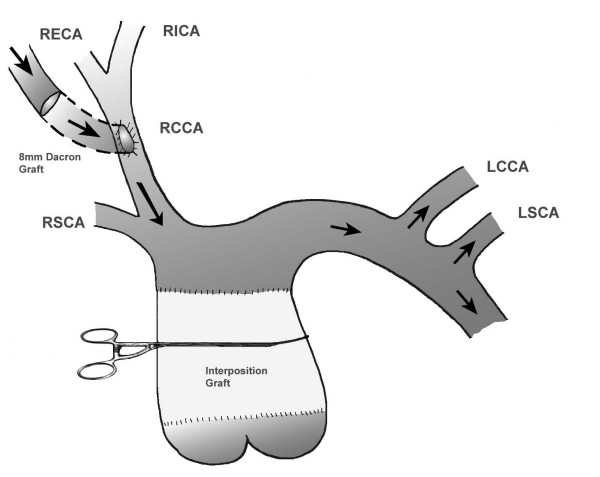
Shows a 8 mm dacron graft sewn onto the right common carotid artery so that the blood is directed towards the aortic arch. Arrows show the direction of the blood. Key: RSCA- right subclavian artery, LSCA- left subclavian artery, RCCA- right common carotid artery, LCCA- left common carotid artery, RECA- right external carotid artery, LECA- left external carotid artery.

Various routes for cannulation for cardiopulmonary bypass (CPB) were discussed due to previous sternotomy and the X-ray finding of close proximity of the repaired ascending aorta to the sternum. During the first operation, an attempt was made to expose the right axillary artery but the artery was not accessible. CPB was established via the right common femoral artery (CFA). The left CFA was not a viable alternative in view of the dissection flap extending down to the left common iliac artery. It was therefore elected to institute CPB through the right common carotid artery (CCA). The right CCA and the left sapheno-femoral junction were exposed. A longitudinal incision was made along the medial border of the sternocleidomastoid muscle at the level of the thyroid cartilage. The internal jugular vein (IJV) and common carotid artery (CCA) were exposed. Two slings were passed behind the CCA to aid the control of the artery for the subsequent steps of the procedure. Skin incision and preparations for the sternotomy were made. Heparin was administered to maintain an activated clotting time (ACT) greater than 400 seconds. Once this was achieved a curved clamp was applied to the exposed carotid artery and a longitudinal 2 cm incision was made. A dacron graft was then sewn onto this incision in such a fashion so as to direct the inflow of the blood towards the aortic arch (figure 1). The arterial cannula for CPB was connected to the other end of the graft. The venous cannula was then introduced into the left femoral vein (figure 1) via the left sapheno-femoral junction using the Seldinger technique. CPB was instituted with careful monitoring of the head for any evidence of oedema or petechial haemorrhage. Patient was gradually cooled to a core temperature of 32°C initially and then to 28°C. Sternotomy was then performed followed by exposure of the heart and the ascending aorta. The native aortic valve was exposed by making an incision in the previous interposition graft to the ascending aorta. The aortic valve was excised and replaced by a size 25 mm mechanical valve. The aortotomy was then closed. Haemostasis was achieved and the patient was weaned successfully off the CPB machine. The CCA incision was closed with 4/0 prolene followed by skin closure. Once the venous cannula was taken out the great saphenous vein was tied off. Postoperatively, no haemodynamic support was required and the patient was discharged after 6 days with no evidence of neurologic, cardiac or renal impairment.

## Discussion

A redo sternotomy is challenging procedure when the heart and the aorta are closely adhered to the back of the sternum. In order to avoid damage to the cardiac chambers or the aorta during redo sternotomy, CPB can be established by peripheral cannulation. Femoral artery and vein cannulation was introduced in the 1960s to achieve circulatory arrest in patients undergoing intracranial operations [1]. This technique has been used successfully in cardiothoracic surgery to establish reliable CPB before a redo cardiac operation. However, femoral artery cannulation in aortic dissection may lead to visceral malperfusion and retrograde embolisation. An option in this setting may be axillary artery cannulation [2,3]. Reported advantages include lack of retrograde embolization, absence of visceral malperfusion and establishment of cerebral blood flow during circulatory arrest [2-5].

In the setting of surgery on the thoracic aorta requiring total CPB and deep hypothermic circulatory arrest (DHCA), extrathoracic cannulation of the left CCA has been reported [6]. However, CCA cannulation for total CPB without DHCA has not been described in adult patients. In our patient with previous sternotomy for repair of type A aortic dissection and radiographic evidence of adherence of heart and aorta to the back of the sternum, establishment of CPB prior to the sternotomy was crucial. Right CFA cannulation would have been difficult due to previous lymphocoele following arterial cannulation to establish CPB. Left CFA cannulation would have potentially resulted in cannulating the false lumen of the dissection. The dissection flap was extending from the aortic root to left common iliac artery. Right axillary artery cannulation was not an option as an unsuccessful attempt at cannulation was made during the first operation. Another possible site is the innominate artery [7], but this would have required a sternotomy first. Thus, right CCA was used together with the left femoral vein to establish CPB prior to redo sternotomy. In order to use the CCA as the sole inflow during CPB, it is vital to ensure that the direction of the inflowing blood is towards the aortic arch rather than the head. This was achieved by sewing the dacron graft onto the CCA at an angle shown in figure 1.

## Conclusion

In circumstances where it is not possible to use alternate arterial cannulation sites like the CFA and axillary artery in redo cardiac surgery in adults, CPB can be safely established via the CCA.

## Competing interests

The author(s) declare that they have no competing interests.

## Authors' contributions

SB and HV were involved in the writing of the report while AH and QA corrected and finalised the manuscript and were the first surgeons. All authors read and approved the final manuscript.
